# Differential Activation of Human Monocytes and Lymphocytes by Distinct Strains of *Trypanosoma cruzi*

**DOI:** 10.1371/journal.pntd.0003816

**Published:** 2015-07-06

**Authors:** Luísa M. D. Magalhães, Agostinho Viana, Egler Chiari, Lúcia M. C. Galvão, Kenneth J. Gollob, Walderez O. Dutra

**Affiliations:** 1 Laboratório de Biologia das Interações Celulares, Departamento de Morfologia, Instituto de Ciências Biológicas, Universidade Federal de Minas Gerais, Belo Horizonte, Minas Gerais, Brazil; 2 Laboratório de Biologia do *Trypanosoma cruzi* e doença de Chagas, Departamento de Parasitologia, Instituto de Ciências Biológicas, Belo Horizonte, Minas Gerais, Brazil; 3 Universidade Federal do Rio Grande do Norte, Natal, Rio Grande do Norte, Brazil; 4 Instituto Nacional de Ciência e Tecnologia em Doenças Tropicais (INCT-DT), Belo Horizonte, Minas Gerais, Brazil; 5 Programa de Pós-Graduação em Medicina e Biomedicina, Instituto de Ensino e Pesquisa, Hospital Santa Casa, Belo Horizonte, Minas Gerais, Brazil; Universidad Autónoma de Yucatán, MEXICO

## Abstract

**Background:**

*Trypanosoma cruzi* strains are currently classified into six *discrete typing units* (DTUs) named TcI to VI. It is known that these DTUs have different geographical distribution, as well as biological features. TcI and TcII are major DTUs found in patients from northern and southern Latin America, respectively. Our hypothesis is that upon infection of human peripheral blood cells, Y strain (Tc II) and Col cl1.7 (Tc I), cause distinct immunological changes, which might influence the clinical course of Chagas disease.

**Methodology/Principal Findings:**

We evaluated the infectivity of CFSE-stained trypomastigotes of Col cl1.7 and Y strain in human monocytes for 15 and 72 hours, and determined the immunological profile of lymphocytes and monocytes exposed to the different isolates using multiparameter flow cytometry. Our results showed a similar percentage and intensity of monocyte infection by Y and Col cl1.7. We also observed an increased expression of CD80 and CD86 by monocytes infected with Col cl1.7, but not Y strain. IL-10 was significantly higher in monocytes infected with Col cl1.7, as compared to Y strain. Moreover, infection with Col cl1.7, but not Y strain, led to an increased expression of IL-17 by CD8+ T cells. On the other hand, we observed a positive correlation between the expression of TNF-alpha and granzyme A only after infection with Y strain.

**Conclusion/Significance:**

Our study shows that while Col cl1.7 induces higher monocyte activation and, at the same time, production of IL-10, infection with Y strain leads to a lower monocyte activation but higher inflammatory profile. These results show that TcI and TcII have a distinct immunological impact on human cells during early infection, which might influence disease progression.

## Author Summary

Chagas disease remains a major public health problem in Latin America with over 13 million people infected. It is believed that the host immune response and genetic diversity of the parasite play an important role in the progression of Chagas disease, which presents a variety of clinical forms ranging from indeterminate to cardiac and digestive forms. Since parasite genetic diversity may influence the development of Chagas disease, our study aims to understand the immune response of human peripheral blood cells upon infection with two *T*. *cruzi* strains with different genetic backgrounds (Col cl1.7 – Tc I, and Y strain – TcII). Our study showed differences in the expression of cytokines and activation molecules between cells infected with strains from Tc I (Col cl1.7) and Tc II (Y strain). These data show the importance of parasite strain in the development of the host response early in infection, which may influence the clinical progression of Chagas disease.

## Introduction

Human infection with the protozoan parasite, *Trypanosoma cruzi*, leads to Chagas disease, which presents as a spectrum of clinical forms, ranging from a relatively mild form (indeterminate), to a severe heart disease that affects approximately 30% of the infected individuals. Chagas disease is endemic to Latin America and the pathogenesis of Chagas heart disease is not clearly understood. A combination of host genetics, the host immune response and parasite factors seem to play important roles in the process [1] [2].

It has been demonstrated that patients with the indeterminate clinical form of Chagas disease display a predominantly modulatory immune environment, with higher production of the anti-inflammatory cytokine IL-10 [3] [4] [5] and IL-17 [6,7], which are produced by monocytes (IL-10) and T cell subsets (IL-10 and IL-17). On the other hand, the response observed in cardiac patients tends to be more inflammatory, with higher production of TNF-alpha and IFN-gamma, which are correlated with worse cardiac function [5,8].

Despite the clear polarity observed in the immune response of patients with different clinical forms, it is not possible to establish whether this is the primary cause of the development of distinct clinical forms. Genetic diversity of the parasite may have a great influence on different clinical outcomes [1]. Supporting this hypothesis, Vago *et al*. demonstrated that parasites isolated from heart or esophagus of Chagas patients display distinct genetic profiles [9].

*T*. *cruzi* strains are currently classified into six *discrete typing units* (DTU’s) named TcI to VI. It is known that these DTU’s have different biological and geographical features [10]. The DTU I is the most abundant of all *T*. *cruzi* DTU’s in the Americas and can be associated with sylvatic and domestic cycles. Despite its extensive distribution throughout the Americas, cases of Chagas disease caused by strains belonging to DTU I are concentrated in the north of South and Central America, with rare cases in the Southern Cone [11]. The DTU II is mostly associated with the domestic cycle and is mostly associated with chronic Chagas disease in South America [12–15]. After an outbreak of acute Chagas disease in Santa Catarina, Steindel *et al*. identified mixed TcI/TcII patterns in strains derived from *Triatoma tibiamaculata*, while strains isolated from patients were TcII [16]. Moreover, Pena *et al*. observed the selection of TcII strains after a mixed infection with TcI/TcII in murine and human macrophages [17].

Y strain of *T*. *cruzi* was isolated from a human host. Col cl1.7 was cloned from the Colombian strain, which was originally isolated from the blood of a chronic cardiac patient [18] and used in several studies since then [11,19]. These two strains represent the two major genetic groups of *T*. *cruzi*–Tc I (Col cl1.7) and Tc II (Y strain) [10] [19]. It was shown that Col and Y strain lead to different infection outcomes in experimental models. Duz *et al*. showed that dogs infected with Colombiana reach the parasitemia peak later than animals infected with Y strain, and that Y strain triggers a more intense immune response during the acute phase of infection in dogs in comparison with Colombiana strain [20]. Murine infection with Col cl1.7 and JG strain (which is Tc II), showed that animals infected with Col cl1.7 had a milder heart inflammation as compared to the JG strain [19].

Given the influence that the host immune response has on disease outcome, our goal was to determine whether infection by Y strain (Tc II) or Col cl1.7 (Tc I) had different effects on immunological characteristics of human monocytes and lymphocytes, which are key for establishing the immune response during infection. Our data showed that Col cl1.7 and Y strains lead to differential activation of monocytes and T cells, that are correlated with their profile of lower and higher virulence observed in animal models, respectively. Moreover, our data suggests mechanisms explaining how differences in parasite strains can lead to differences in human disease progression and outcome.

## Materials and Methods

### Human samples

The donors included in our studies were non-chagasic healthy individuals (n = 9), as determined by negative specific serological test for Chagas disease. Individuals were from Belo Horizonte city, state of Minas Gerais, Brazil, with ages ranging between 23 and 34 years (average ± SD: 27±4.2). Five donors were males and 4 were females. We excluded from our study individuals with any chronic inflammatory disease, diabetes, heart and circulatory illnesses (including hypertension) or bacterial infections. All individuals included in this work were volunteers and provided written informed consent. This work was approved by the Ethical Committee of the Universidade Federal de Minas Gerais, under the protocol# ETIC077/06. Peripheral blood was collected from the donors by venipuncture.

### Parasites

Tissue culture derived trypomastigotes (TCT) of the Y strain and Col cl1.7 were isolated from infected monolayers of Vero cells. Vero cells were infected using five TCT/host cells and kept in RPMI enriched with 5% inactivated fetal calf serum (FCS), supplemented with antibiotics (penicillin at 500μ/mL and streptomycin at 0.5 mg/mL). After approximately 5 days, the TCT were collected from the supernatant, washed once by centrifugation with phosphate-buffered saline (PBS) pH 7.2 at 1000g for 10 min at 4°C and resuspended in RPMI to 6x10^7^ TCT/mL. Parasites obtained in such manner were used for infecting adherent cells and peripheral blood cells from donors.

### Adherent cell preparation, infection and confocal analysis

Adherent cells were used solely to confirm the infectivity of monocytes by the different strains using confocal microscopy. Peripheral blood mononuclear cells (PBMC) were purified as previously done by us [5]. Briefly, heparinized blood was diluted 1:1 with PBS and applied over a Ficoll gradient. The mixture was centrifuged for 40 min at 600g. PBMCs were collected at the interface between the plasma and the Ficoll. Cells were washed three times by centrifugation with PBS and resuspended in complete RPMI (RPMI supplemented 5% of human sera, antibiotics—penicillin at 500U/ml and streptomycin at 0.5 mg/ml—and 1mΜ of L-glutamine). To obtain adherent cells, 2x10^6^ PBMC/well were plated on 13-mm round coverslips in complete RPMI and incubated for 3 hours at 37°C, 5% CO_2_. After incubation, non-adherent cells were removed by washing the wells with warm PBS and the adherent cells (monocytes) were used in infection experiments as described below. As previously determined by us, adherent cells obtained using this protocol are approximately 85% Cd11b+ or CD14+ [5].

The infection of monocytes (adherent cells) was performed as previously done by us [5]. Briefly, infection was performed over coverslips in duplicates. Parasites from Y or Col cl1.7 were added at a ratio of 10:1 TCT/monocytes and incubated for 3 hours. After the incubation period the monolayers were washed with PBS to remove extracellular parasites and re-incubated for 12 or 69 hours in complete RPMI, completing a total of 15 and 72 hours of culture. At the end of the culture time, cells were fixed by incubating the slides with 300ul of paraformaldehyde for 60 minutes at room temperature, washed three times with PBS and immunofluorescence was carried out by staining with 4’6’-diamino-2-phenylindole (DAPI). Briefly, coverslips containing the infected adherent cells were incubated with DAPI diluted 1:300 in PBS for 15 min at room temperature and mounting using Vectashield (Vector laboratories). Confocal analyses were performed using a Meta-510 Zeiss laser scanning confocal system running LSMix Software (Oberkochen, Germany) coupled to a Zeiss microscope using an oil immersion Plan-Apochromat objective (63X, 1.2 numerical aperture, Oberkochen, Germany).

### Infection of peripheral blood cells

Infection of whole blood was used for all experiments of surface molecule and cytokine expression analysis. For the infection of peripheral blood cells, trypomastigotes from Vero cultures, obtained as described above, were labeled with CFSE (carboxyfluorescein diacetate succinimidyl ester–Molecular Probes C1157) by using a protocol previously reported by us [5], with modifications. Briefly, 6.0 x 10^7^ parasites were incubated with 5μM CFSE for 15 min at 37°C under 5% CO_2_. Labeled parasites were washed three times with cold PBS + 10% of inactivated fetal bovine serum by centrifugation at 1000g for 10 min at 4°C.

The infection was performed using 10 parasites/cell. Cells and parasites were incubated in suspension at 37°C in 5% CO_2_ for 3 hours with complete RPMI. After the incubation period the cells were washed by centrifugation with PBS at 600g for 10 min at 4°C to remove extracellular parasites. For the incubation of “15 hours” and “72 hours” we re-incubated the cultures for additional 12 and 69 hours respectively, after washing off the free parasites. Brefeldin A (1μg/ml) was added for the last four hours of infection in both groups (15 and 72 hours) to prevent protein secretion.

### Analysis of expression of surface molecules and cytokines by peripheral blood cells using flow cytometry

**Fig 1:**
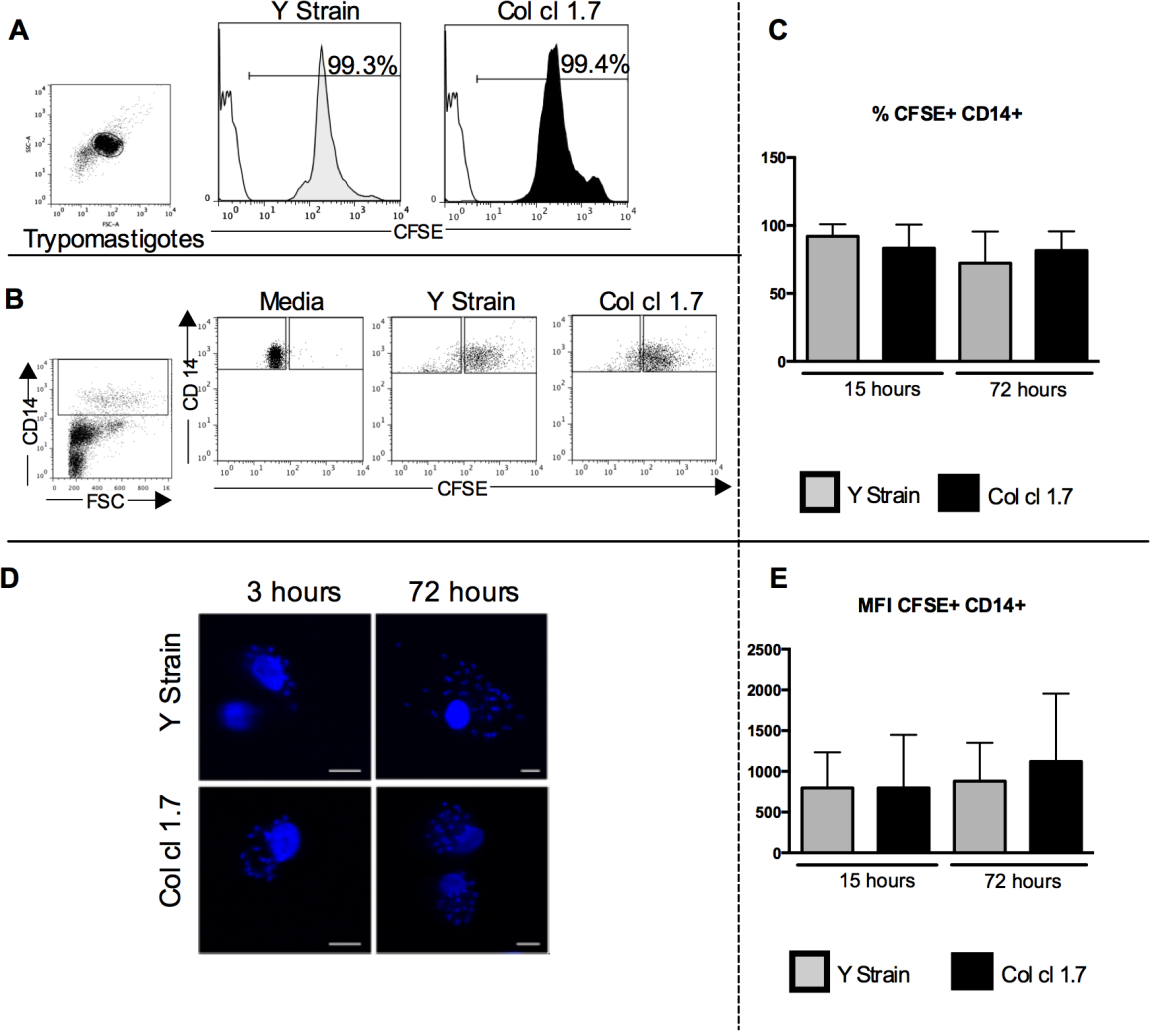
Evaluation of the frequency and intensity of *T*. *cruzi* infection in human monocytes. Results are expressed as average ± standard deviation. The symbol * indicates *p* < 0.05 between groups. Trypomastigotes from Y strain and Col cl1.7 were previously stained with CFSE and used for infections for 15 and 72 hours. (A) Representative figure of CFSE staining on trypomastigotes of Y strain and Col cl1.7. The plot shows the population of trypomastigotes; the white peaks in the histograms represent isotype control and the gray and black peaks represent Y and Col cl1.7 strains, respectively. (B) Representative figure of gating strategy for defining the CFSE+ and CFSE- in CD14+ cells, showing the first gate considering size (FSC) versus CD14 expression, followed by the gates considering CD14+ and CFSE+ and CFSE-. (C) Frequency of CFSE+ CD14+ cells. (D) Representative confocal microscopy analysis, showing DAPI-stained parasite’s nuclei inside monocytes. Cells were infected with Y strain or Col cl1.7 for 15 or 72 hours with 10 parasites/cell, preparation were stained with DAPI, and read on a confocal microscope, as described in material and methods; magnification x62. (E) Mean intensity of expression of CFSE fluorescence.

After 15 and 72 hours of incubation, the erythrocytes were lysed using RBC “Lysing buffer” (Bio Legend) at 20mL/1mL of peripheral blood. The tubes were incubated for 15 min at 20°C in the dark. After the incubation, cells were washed three times with PBS by centrifugation at 600g for 10 min at 4°C and resuspended in PBS to 10^7^cells/ml. Cells were then immunostained and analyzed using multiparametric flow cytometry. 200.000 cells were incubated for 15 min at 4°C with different antibody combinations. Samples were washed three times in PBS-1% bovine serum albumin (BSA) and fixed by 20-min incubation with 2% formaldehyde solution. After removal of the fixation solution by centrifugation and washing once with PBS, we permeabilized the cells by incubation for 10 min with 0.5% saponin solution, centrifuged and incubated with antibodies to intracellular molecules for 30 min at 20°C. The antibodies to surface molecules used were: anti-CD4, anti-TLR-2 or anti-CD69 – labeled with PE; anti-CD14 – labeled with APC; anti-CD8, anti-HLA-DR, anti-CD80 – labeled with PE-Cy7; anti-CD86 – labeled with Pacific Blue and anti-CD4 labeled with APC-Cy7. For intracellular staining we used the following antibodies: anti-TNF-alpha, anti-IL-12/IL-23p40, anti-IL-10 and anti-Granzyme A. All antibodies were purchased from BioLegend, San Diego, CA, USA. After intracellular staining, cells were washed and resuspended in PBS and acquired using a FACSCanto II (Becton & Dickinson, San Jose, CA, USA). A total of 30,000 lymphocyte events were acquired and the parameters were analyzed in the monocyte or lymphocyte population. Lymphocyte analysis was done by gating the region occupied classically by lymphocytes in a size *versus* granularity plot, followed by gating in CD4+ or CD8+ cells. For monocytes, we first gated on CD14high cells in plot of size versus CD14 and further gated on CD14+CFSE+, CD14+CFSE- (Fig 1B). The analyses were performed using FlowJo 7.6.5 software (Tree Star Inc., Ashland, OR, USA).

### Statistical analysis

We compared our results using One-Way Anova or Kruskal-Wallis test according to Kolmogorov-Smirnov normality test. All samples were submitted to rout test to identify outliers. Correlation analyses were made using Pearson’s correlation coefficient. All analyses were performed using Graph Pad Prism Software (La Jolla, CA, USA). Differences that returned *p* values equal or less than 0.05 were considered statistically significant from one another.

## Results

### The frequency and intensity of infection of human monocytes by Y strain and Col cl1.7 trypomastigotes was similar

To determine the rate of infection with Y strain or Col cl1.7, we stained *T*. *cruzi* trypomastigotes with CFSE and infected peripheral blood cells from healthy donors, as described above. It is known that *T*. *cruzi* infects primarily monocytes [5], thus we analyzed the CD14+CFSE+ cells, which corresponds to the monocytes that were infected with trypomastigotes. Fig 1A shows that the efficiency of labeling parasites from Y or Col 1.7 strains with CFSE is similar and Fig 1B shows the gating strategy for CFSE+ cells (infected) as well as CFSE- cells (non-infected) that was used in our analysis of surface molecule and cytokine expression.

We observed a similar frequency of CD14+CFSE+ cells when the infection was performed with Y or Col cl1.7, after 15 or 72 hours of culture, comparing the different strains (Fig 1C). The intensity of infection on a cell per cell basis was also similar between the cells infected with the different strains (Fig 1D). Fig 1E shows representative analysis using fluorescent confocal microscopy, depicting infection of monocytes by parasites of either isolates stained with DAPI, confirming monocyte infection.

### Y strain or Col 1.7-infected monocytes express higher intensity of HLA-DR and TLR-2

**Fig 2:**
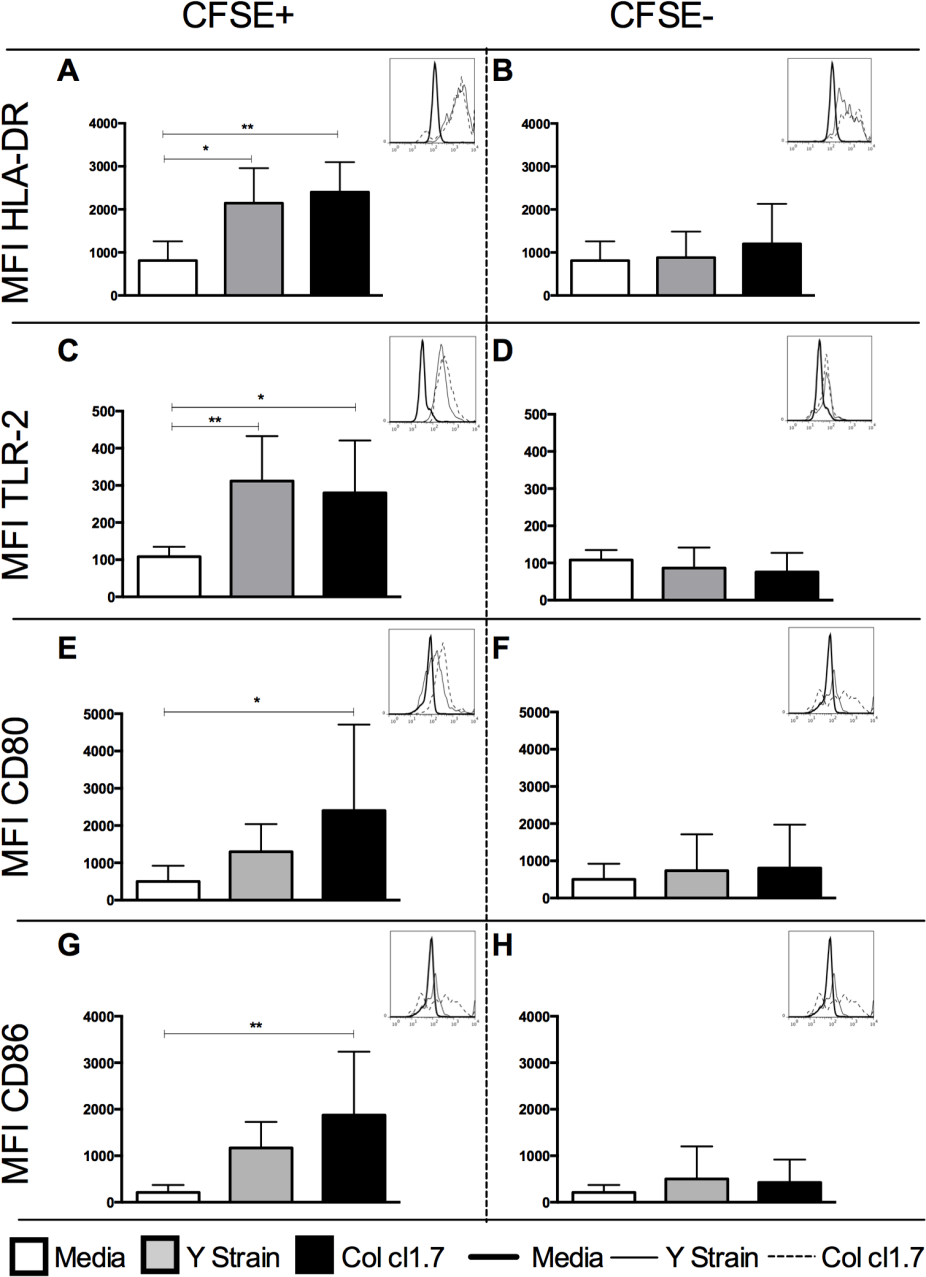
Determination of the MFI of HLA-DR, TLR-2, CD80 and CD86 in human monocytes infected or not with strains of *T*. *cruzi*. Results are expressed as average ± standard deviation. The symbol * indicates *p*< 0.05 between groups. Determination of mean intensity of (A) HLA-DR in CFSE+ monocytes after 15 hours of culture; (B) HLA-DR in CFSE- monocytes after 15 hours of culture; (C) TLR-2 in CFSE+ monocytes after 15 hours of culture; (D) TLR-2 in CFSE- monocytes after 15 hours of culture; (E) CD80 in CFSE+ monocytes after 72 hours of culture; (F) CD80 in CFSE- monocytes after 72 hours of culture; (G) CD86 in CFSE+ monocytes after 72 hours of culture; (H) CD86 in CFSE- monocytes after 72 hours of culture. Histogram inserts show a representative overlay for the expression of each of the molecules under the different conditions.

In order to access if there was a difference in monocyte activation after the infection with Y strain or Col cl1.7, we analyzed the expression of HLA-DR and TLR-2. HLA-DR is an important antigen-presenting molecule whose expression changes upon activation [21]. It is known that activation of TLR-2 is involved with activation of Rab-5, fusion with endosomes and phagocytosis of the trypomastigote form [22]. Since these changes occur early after activation, we evaluated the expression of HLA-DR and TLR-2 after 15 hours of infection. Our results show that monocytes infected with either *T*. *cruzi* strain (CD14+CFSE+ cells) express higher intensity of HLA-DR and TLR-2 compared with media control (Fig 2A and 2C). On the other hand, non-infected monocytes (CD14+CFSE- cells) did not show significant changes in the intensity of expression of these molecules as compared to media control (Fig 2B and 2D).

### Infection with Col 1.7 led to higher expression of CD80 and CD86 by infected human monocytes

We investigated whether the expression of CD80 and CD86, monocyte ligands for co-stimulatory molecules, was modified after 72 hours of infection with Y strain or Col cl1.7. We also evaluated the expression of these molecules after 15 hours of infection and, although the results showed a similar trend as the ones observed in 72 hours, the frequencies and intensities were much lower (S1 Fig), indicating that the kinetics of expression of these molecules seems to be slower in response to *T*. *cruzi* infection. Thus, we chose to perform all analysis after 72 hours. Our results showed that CD14+CFSE+ monocytes infected by Col cl1.7 showed an increase in the intensity of expression of both co-stimulatory molecules, CD80 and CD86, as compared to media control (Fig 2E and 2G). Infection with Y strain did not have a significant effect on the expression of CD80 or CD86. Expression of CD80 or CD86 did not change in non-infected monocytes (CD14+CFSE-) (Fig 2F and 2H).

### Y strain or Col cl1.7 infection of monocytes induces expression of IL-12, TNF-alpha+ and IL-10+, however Col cl1.7 infection induces greater IL-10 production as compared to Y strain

**Fig 3:**
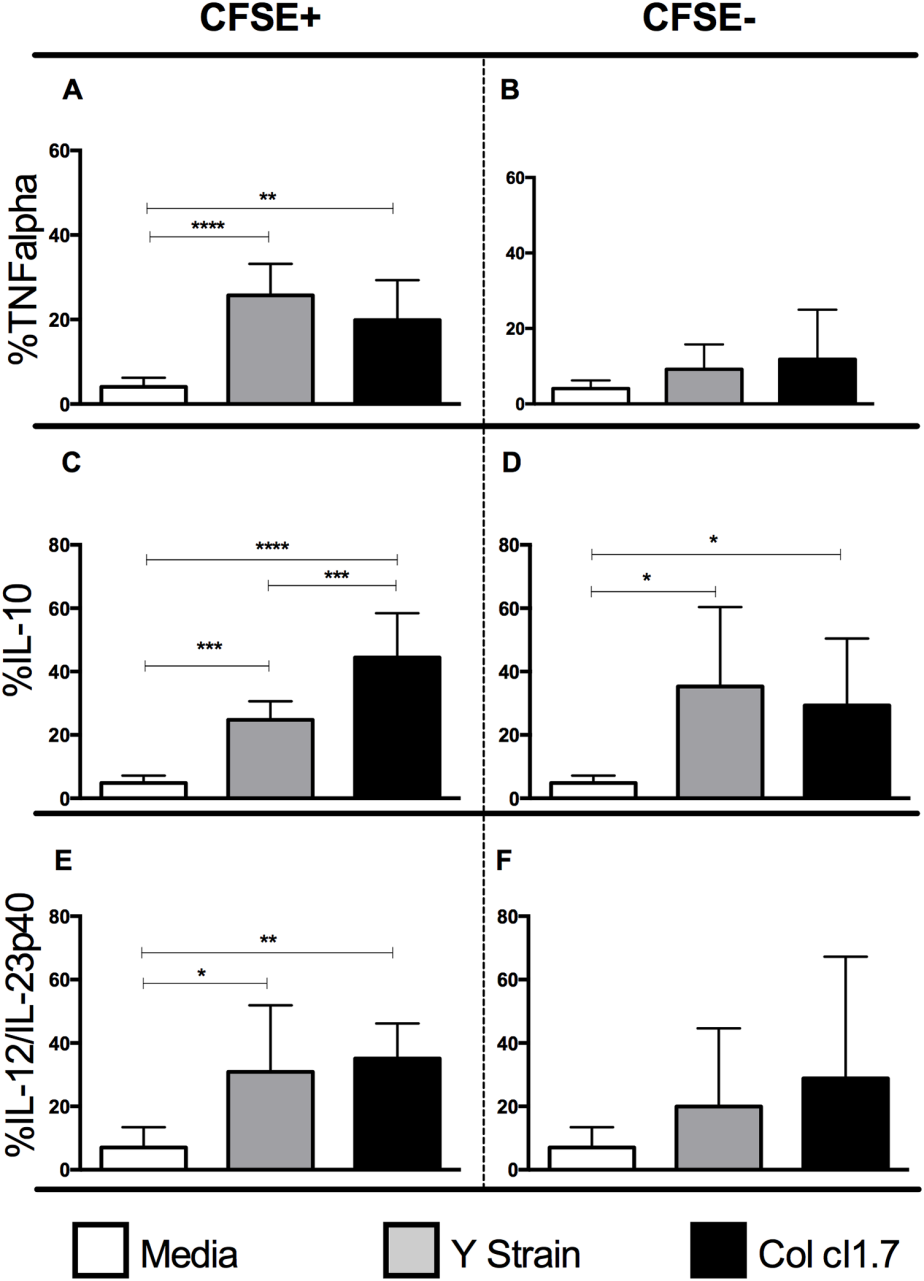
Determination of the percentage of expression of the cytokines TNF-alpha, IL-12/IL-23p40 and IL-10 by human monocytes infected or not with different strains of *T*. *cruzi*. Results are expressed as average ± standard deviation. The symbol * indicates *p* < 0.05 between groups. Determination of percentage of (A) TNF-alpha in CFSE+ monocytes; (B) TNF-alpha in CFSE- monocytes; (C) IL-12/IL-23p40 in CFSE+ monocytes; (D) IL-12/IL-23p40 in CFSE- monocytes; (E) IL-10 in CFSE+ monocytes; (F) IL-10 in CFSE- monocytes; All analysis done after 15 hours of culture.

We questioned whether the monocyte activation triggered by infection had an influence on the expression of cytokines after 15 hours of infection with the Y strain or Col cl1.7. We observed a higher expression of TNF-alpha and IL-12/IL-23p40 in monocytes infected with the two strains compared to media control (Fig 3A and 3C, respectively). No changes were observed in non-infected monocytes (Fig 3B and 3D).

When we analyzed the expression of the anti-inflammatory cytokine IL-10 (Fig 3E and 3F), we observed an increase in infected and non-infected cells as compared to media, regardless of the strain. However, we observed a higher percentage of IL-10+ cells when cultures were infected by Col cl.1.7, compared to infection by the Y strain (Fig 3E).

### Y strain or Col cl1.7 infection of monocytes induces higher intensity of activation molecule expression and cytokines when compared to non-infected monocytes

We then evaluated the intensity of molecule expression by infected (CD14+CFSE+) and non-infected (CD14+CFSE-) monocytes exposed to either Y strain or Col cl1.7. Interestingly, it was observed that monocytes infected by Y strain or Col cl1.7 (CD14+CFSE+) had a more intense expression of HLA-DR, TLR2, CD80, CD86, IL-12/IL-12p40, TNF-alpha and IL-10 as compared to monocytes in the same cultures that were not infected by the parasite (CD14+CFSE-) (Table 1). These data suggests that direct contact and infection by the parasite is important to induce the phenotypic and functional changes seen in the infected cultures and that some changes in molecule expression in non-infected cells may be due to bystander effects.

**Table 1:** Analysis of the mean intensity of expression of surface molecules and cytokines in monocytes CFSE+ and CFSE- after 15 hours of infection with Y strain and Col cl1.7.

	Y strain			Col cl 1.7	
	CFSE+	CFSE-		CFSE+	CFSE-
**HLA-DR**	2146±813^a^	883±604 ^a^	**HLA-DR**	2400±697 ^b^	1202±931 ^b^
**TLR-2**	312±121 ^c^	87±55 ^c^	**TLR-2**	280±141 ^d^	76±52 ^d^
**IL12/IL23p40**	604±650 ^e^	51±30 ^e^	**IL12/IL23p40**	802±781 ^f^	43±21 ^f^
**TNF-alpha**	293±187 ^g^	74±59 ^g^	**TNF-alpha**	384±237 ^h^	79±54 ^h^
**IL10**	318±162 ^i^	114±79 ^i^	**IL10**	409±331 ^j^	83±51 ^j^

Cells were double stained for the monocyte marker CD14 and the different molecules. Trypomastigotes from Y strain and Col cl1.7 were stained for CFSE. The analysis was performed by multiparametric flow cytometry, as described in Material and Methods. Results are expressed as the mean intensity of the molecule within the CD14+CFSE+ or CD14+CFSE- subpopulations and are indicated as average ± standard deviation for each analysis. Equal letters represent statistical difference (*p* < 0.05). Comparisons between groups were performed using One-Way Anova or Kruskal-Wallis test according to Kolmogorov-Sminorv normality test.

### Y strain and Col cl1.7 led to higher expression of TNF-alpha and granzyme A by CD8+ T lymphocytes, while only Col cl1.7 infection induced CD8+ IL-17 expressing cells

**Fig 4:**
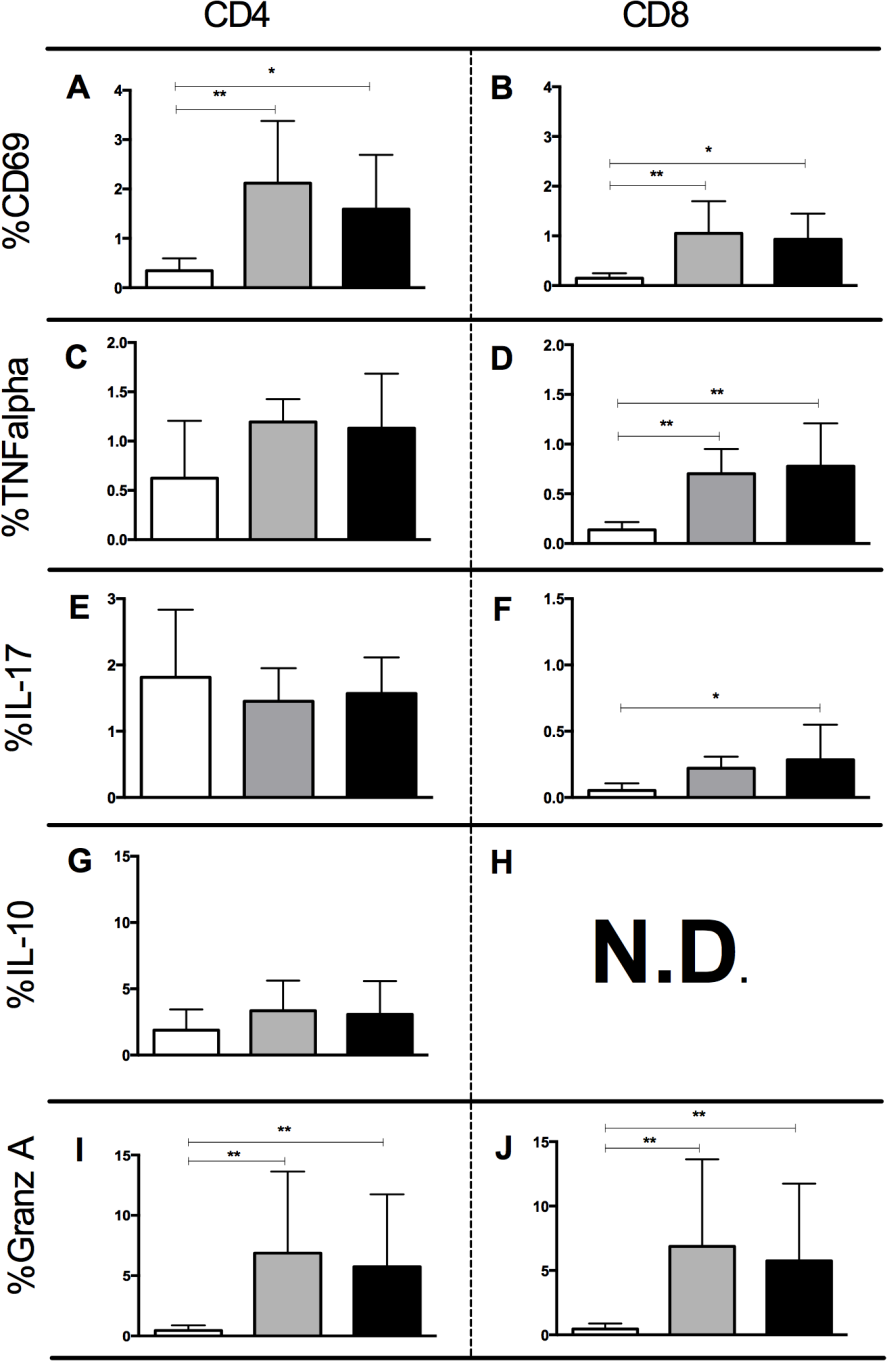
Determination of the percentage of expression of CD69, TNF-alpha, IL-17, IL-10, TNF-alpha and Granzyme A in CD4 and CD8 lymphocytes after exposure to monocytes infected with Y strain and Col cl1.7. Results are expressed as average ± standard deviation. The symbol * indicates *p* < 0.05 between groups. Determination of percentage of (A) CD69+ CD4+ after 72 hours of culture; (B) CD69+CD8+ after 72 hours of culture; (C) TNF-alpha+CD4+ after 72 hours of culture; (D) TNF-alpha+CD8+ after 72 hours of culture; (E) IL-17+CD4+ after 15 hours of culture; (F) IL-17+CD8+ after 15 hours of culture; (G) IL-10+ CD4+ after 72 hours of culture; (H) ND; (I) Granzyme A+CD4+ after 72 hours of culture (J) Granzyme A+CD8+ after 72 hours of culture.

Despite the low frequency of infection by Col 1.7 and Y strains in CD4 and CD8 lymphocytes (supporting material, S2 Fig), these cells have a major role in orchestrating the immune response against the parasite [2] and their activation depends on the interaction with infected monocytes. In order to determine if exposure to monocytes infected with Y strain and Col cl1.7 led to differences in lymphocyte activation, we analyzed the expression of the activation molecule CD69 and cytokines in lymphocytes after 72 hours of infection with both lineages. Our data showed that infection with either lineage led to a higher expression of the activation molecule CD69 by CD4+ and CD8+ T lymphocytes as compared to media control (Fig 4A and 4B).

We then evaluate the expression of the pro-inflammatory cytokine TNF-alpha. This cytokine is known to be important in the control of *T*. *cruzi* during the early stages of infection acting synergistically with IFN-gamma and activating monocytes to produce nitric oxide [23–26]. However, the persistence of a TNF-alpha-rich, pro-inflammatory environment, is associated with pathology during the chronic phase [2]. While no change was observed in the frequency of CD4+TNF-alpha+ T cells after 72 hours of infection with either *T*. *cruzi* strain (Fig 4C), our data showed an increase in the frequency of CD8+TNF-alpha+ T lymphocytes (Fig 4D).

We observed a higher expression of IL-17 in CD4+ T lymphocytes after infection with both strains compared to media control (Fig 4E). However, the increase expression of IL-17 in CD8+ T lymphocytes was observed only after infection with Col cl1.7 (Fig 4F).

IL-10 has a crucial role in orchestrating the immune response during *T*. *cruzi* infection, modulating the immune response in chronic disease [2–4]. The infection with either strain did not alter the percentage of expression of IL-10 by CD4+ T lymphocytes (Fig 4G).

Similar to TNF-alpha expression by CD4+ T cells after 72 hours, infection with either strain did not induce an increase expression of granzyme A (Fig 4I). However, we observed a higher expression of granzyme A by CD8+ T lymphocytes after infection with either strain compared to media control (Fig 4J).

### An increased expression of TNF-alpha is associated with an increase in the expression of Granzyme A but only after infection with the Y strain

**Fig 5:**
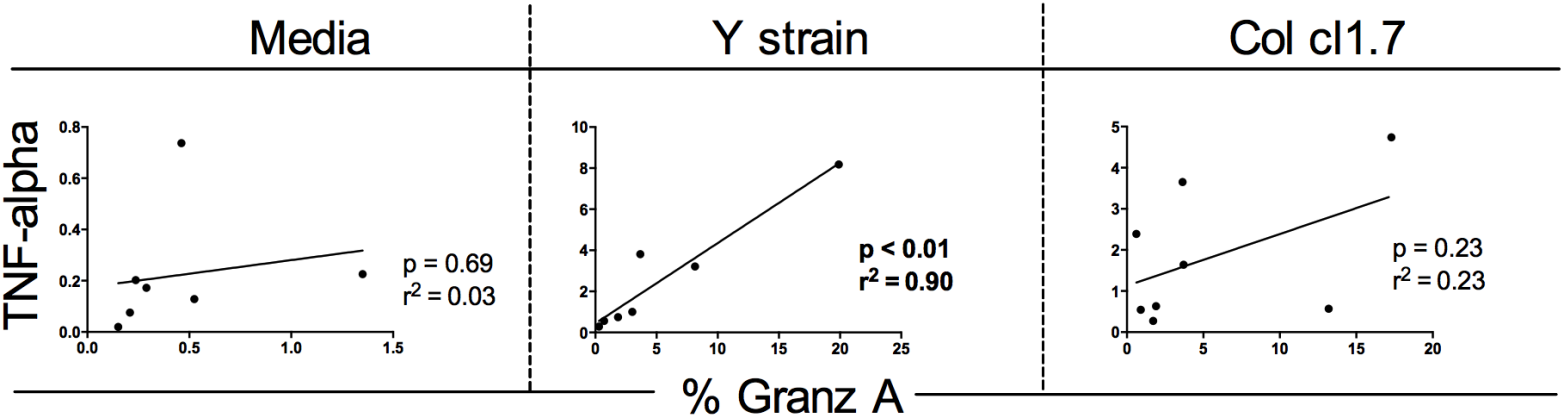
Correlation analysis of the expression of Granzyme A with the expression of TNF-alpha in CD8 T cells. Statistical significance (*p* value) is indicated in each graph together with the r^2^ value.

We performed correlation analysis to determine whether the increase in expression of Granzyme A by CD8 T lymphocytes was associated with the expression of TNF-alpha by these cells. Our data showed a positive correlation between the expression of TNF-alpha and the expression of Granzyme A. Interestingly, this correlation was only observed after the infection with Y strain and not in media or infection with Col cl1.7 (Fig 5).

## Discussion

A triad of factors involving host genetics, immune competence of the affected population, and genetic diversity of the parasite influence the outcome of Chagas disease [1]. Our goal was to evaluate the effects of infection with either the Y (Tc II) or Col cl1.7 (Tc I) strains on immunological characteristics of human peripheral blood cells. These two strains were isolated from humans and represent the two major genetic groups of *T*. *cruzi*–Tc I (Col cl1.7) and Tc II (Y strain) [10] [19].

Our data showed that the frequency of infected cells was similar when comparing Y strain and Col cl1.7. The intensity of infection was measured by CFSE (Fig 1D) and also by DAPI staining (Fig 1E) and showed that the intensity of infection on a cell-per-cell basis did not change when comparing infection with Col cl 1.7 and Y strains. We next questioned if the similar rate of infection with both Y strain and Col cl1.7 could differently activate monocytes. We analyzed the expression of activation-related molecules (HLA-DR, TLR-2, CD80 and CD86) in infected (CFSE+) and non-infected (CFSE-) monocytes, from cultures exposed to the different strains. This separation allows us to determine the effects of direct contact with the parasite versus a bystander effect in the expression of the molecules. We observed that infection with both strains led to a higher intensity of HLA-DR and TLR-2 expression by infected monocytes (CD14+CFSE+), as compared to media controls; these increases of intensity did not occur in non-infected monocytes (CD14+CFSE-) from the same cultures. It is known that *T*. *cruzi* activates TLR-2 through GPI-anchored mucin-like glycoproteins (tGPI-mucin) and this activation triggers an inflammatory response [27]. Moreover, it has been shown that TLR-2 and TLR-9 are important for parasite control in the early phases of infection [28]. It is also known that TLR-2 is involved with the internalization of *T*. *cruzi* in murine macrophages via activation of Rab5 [22]. Thus, the increase in the intensity of TLR-2 only in infected monocytes supports the hypothesis that activation of TLR-2 is important in *T*. *cruzi* phagocytosis [22]. We also investigated the expression of the ligands for co-stimulatory molecules, CD80 and CD86. These co-stimulatory molecules provide the second signal necessary for T cell activation [29] and their expression has also been associated with activation of monocytes after *T*. *cruzi* infection [30]. Surprisingly, while infection with Y strain and Col cl1.7 both led to an increase in the intensity of expression of HLA-DR and TLR-2, the two isolates affected differently the expression of CD80 and CD86. Infection with Col cl1.7, but not with Y strain, led to an increase in the expression of CD80 and CD86. This suggests a higher activation of monocytes after 72 hours of infection when infected by Col cl1.7, as compared to Y strain.

Several studies have reported the importance of macrophage activation in experimental infection with *T*. *cruzi*. In the acute phase, after interaction with *T*. *cruzi*, macrophages produce inflammatory cytokines such as IL-12 and TNF-alpha, which activate the production of IFN-gamma by NK cells. The IFN-gamma produced, together with TNF-alpha, activates macrophages to produce oxygen derivatives to eliminate the parasite [23–26]. On the other hand, the anti-inflammatory cytokine IL-10 appears to be detrimental in the early infection. Experimental data suggest that the expression of anti-inflammatory cytokines inhibit IFN-gamma, decreasing the trypanocidal activity of macrophages [31,32]. However, the maintenance of a phenotype with high expression of inflammatory cytokines compared to the expression of anti-inflammatory cytokines is associated with progression to the cardiac clinical form during the chronic phase of human disease [2]. Our data showed an increase in the percentage of TNF-alpha+ monocytes after the infection with Y strain and Col cl1.7. This increase was observed only in infected monocytes, and the intensity of expression of TNF-alpha was also greater in infected monocytes compared to bystander cells.

The percentage of monocytes expressing IL-10 was higher after the infection by the two strains in infected and non-infected monocytes. However, an even higher percentage in IL-10+ monocytes was observed in cultures infected with Col cl1.7, as compared to Y strain. It is known that the expression of IL-10 is crucial for the orchestration of the immune response during chronic Chagas disease. A higher frequency of monocytes producing IL-10 can be found in indeterminate as compared to cardiac patients [5]. Interestingly, in this study, the expression of IL-10 in monocytes was the only parameter that allowed for distinguishing between the two strains, where we observed a higher IL-10 expression after infection with Col cl1.7 compared to infection with Y strain. This result shows that although monocytes infected with both strains show similar activation, the production of cytokines differ between them. The higher expression of IL-10 by monocytes infected with Col cl1.7 suggests that this isolate is able to induce a more balanced immune response, as compared to Y strain. This is consistent with the fact that Col induces a more mild infection than does Y strain in experimental models [19] [20]. This is an important finding, as it suggests that strains of parasite that are able to induce a more balanced response, with TNF-alpha and IL-10 production, may lead to a better infection outcome. The fact that a balanced immune response may be beneficial is supported by the findings that showed that while cardiac patients have a predominantly inflammatory profile (higher expression of TNF-alpha and IFN-gamma), indeterminate patients, despite producing inflammatory cytokines, have a more anti-inflammatory profile (due to high IL-10 expression) [33] [5].

It is known that IL-12/IL-23p40 triggers the expression of IFN-gamma, which is important for the control of *T*. *cruzi* in experimental models [34]. We observed an increased expression of IL-12/IL-23p40 only in infected monocytes after infection with both strains, indicating activation, and suggesting that the production of this cytokine early on may be critical for parasite control, regardless of the strain.

Our data demonstrated a higher activation of infected monocytes as compared to non-infected cells in the same cultures, suggesting that a direct contact with the parasite is required for better activation. This was confirmed by the fact that cells infected by either of the two strains have more intense expression of HLA-DR, TLR-2, CD80, CD86, as well as cytokines IL-12/IL23p40, TNF-alpha and IL-10 when compared with bystander cells. Carvalho and colleagues in 2008 observed that dendritic cells (DCs) infected with *Leishmania braziliensis* were the main cell type responsible for the expression of TNF-alpha, while bystander DCs have enhanced HLA-DR expression [35]. Our results do not corroborate these findings, showing that monocytes infected with two strains of *T*. *cruzi* exhibit greater activation and are also the main producers of cytokines compared to bystander cells. Thus, *T*. *cruzi*-infected monocytes appear to be responsible for the activation of lymphocytes and cytokine expression, while bystander cells appear to be less responsive.

The presence of activated T cells has been observed in the endomyocardial tissue and peripheral blood of patients infected with *T*. *cruzi* in acute and chronic phase of Chagas disease [36] [37] [38]. Given the observed activation of monocytes and their importance in T cell activation, we analyzed the activation state and the expression of cytokines and granzyme A by CD4+ and CD8+ T cells. We first showed that CD4+ and CD8+ T lymphocytes display higher expression of CD69 after infection with Y strain and Col cl1.7, showing that both strains induce T cell activation. We next analyzed the expression of cytokines to determine if the activation was also correlated with cytokine production. No difference in IL-10 expression was seen after infection with either *T*. *cruzi* strains in CD4+ T cells, as compared to media control. However, expression of TNF-alpha by CD8+ T lymphocytes was increased following infection with both strains. The same happened with the expression of Granzyme A. It is not surprising that CD8+ T cells seem to be more functionally responsive to the phenotypic and functional changes than CD4+ T cells, considering that the stimulation was performed with live trypomastigotes, which favors activation via class I molecules, since *T*. *cruzi* is an intracellular parasite [39]. Despite that, activation via class II is also possible, since soluble proteins of the parasite, like TS proteins, are able to activate CD4+ T lymphocytes, which corroborates with the higher expression of CD69 also observed in CD4+ cells [40].

A protective role of IL-17 in experimental *T*. *cruzi* infection was recently reported [41] [42]. In chronic chagasic patients, high levels of IL-17 are related to better clinical prognosis [6,7]. Here we observed a higher expression of IL-17 in CD4+ T cells after the infection with both strains. Interestingly, only the infection with Col cl1.7 led to a higher expression of IL-17 in CD8+ T lymphocytes. Erdmann et al. observed that IL-17 stimulates macrophages to phagocyte trypomastigotes, trapping *T*. *cruzi* in endosomal/lysosomal compartments and enhancing exposure time to antimicrobial effectors of the macrophages that subsequently led to eradication of parasites [43]. The fact that IL-10 and IL-17 both have been associated with the indeterminate form [5] [7] suggests that the induction of IL-17 by Col cl1.7 may also be associated with the more mild infection observed in experimental models, as compared to Y strain.

Expression of TNF-alpha and Granzyme A also increase only in CD8+ T cells. The increased expression of IL-17, TNF-alpha and Granzyme A in CD8+ T cells was an interesting finding, since several data have shown an important role for CD8+ T cells in murine models, as well as in human disease [44] [45]. Interestingly, it has been shown that Granzyme A *knockout* mice are more susceptible to infection by *T*. *cruzi* [46], suggesting a role for this molecule in control of parasitemia. Moreover, expression of granzyme is high in CD8+ T cells found in the inflammatory infiltrate of patients with severe chronic chagasic cardiomyopathy, which is also rich in TNF-alpha+ cells [36]. We then asked whether the expression of the cytotoxic molecule Granzyme A by CD8 T cells was related to the expression of TNF-alpha. We observed a positive correlation between the expression of TNF-alpha and the expression of Granzyme A only after the infection with Y strain.

Taken together, our results show that infection of human cells with the Col cl1.7 leads to a higher expression of CD80 and CD86, as well as of IL-17, favoring monocyte activation. In addition, the observed higher expression of IL-10 by cells infected with Col cl1.7 might be important to avoid tissue pathology in the acute infection, favoring host survival. These data are consistent with the results observed in experimental models, in which Col parental strain and its clones displays low virulence and are able to induce chronification of infection [19,20]. On the other hand, Y strain led to a more inflammatory profile with high TNF and Granzyme expression, which might be associated with pathology. This is also consistent with the high virulence data observed in experimental models [20]. An important data is that TcI and TcII are found more frequently in north and south of Latin America, respectively, and pathology associated with Chagas disease is more frequent in southern than northern Latin America [1]. While a clear association between TcII and greater pathology was not directly performed, this suggests that TcII is at least more frequent in an area with more pathology than TcI.

Transcritome analysis of myoblast cell line infected with different strains of *T*. *cruzi* belonging to Tc I and Tc II, showed that different strains lead to several changes in gene transcription, and that these changes were significantly different amongst strains [47]. Interestingly, the Y strain, also used in our study, led to the least changes in the myoblasts transcriptome profile [47]. The results presented here show, for the first time, the mechanisms by which Y and Col cl1.7 strains influence differently the host’s immune response, while clearly showing the importance of the parasite strain in shaping the host response early on, which might influence disease outcome at later times. Thus, analysis of these parameters in individuals with acute infection of Chagas disease might bring valuable information for patient follow up and care.

## Supporting Information

S1 Fig Determination of the MFI of CD80 and CD86 in human monocytes infected or not with strains of *T*. *cruzi*. Results are expressed as average ± standard deviation. Determination of mean intensity of (A) CD80 in CFSE+ monocytes after 15 hours of culture; (B) CD80 in CFSE- monocytes after 15 hours of culture; (C) CD86 in CFSE+ monocytes after 15 hours of culture; (D) CD86 in CFSE- monocytes after 15 hours of culture.
(TIFF)

S2 Fig Analysis of the infectivity of CD4+, CD8+ and CD14+ cells by Y strain and Col cl.1.7. Representative dot-plots of the analysis of infection of CD4 and CD8+ cells (gated on lymphocytes) and CD14+ cells (gated on monocytes) by the different isolates. The first three panels show media control and the others show Y strain and Col cl1.7, for each cell population. The figure shows that CD4+ and CD8+ cells display very low infection by both strains and that monocytes are the main infected cell population. Moreover, no differences were observed when comparing monocyte infectivity by Y and Col cl1.7 isolates (quantitative data shown in Fig 1C and 1D).
(TIFF)

## References

[1] Buscaglia CA, Di Noia JM (2003) Trypanosoma cruzi clonal diversity and the epidemiology of Chagas' disease. Microbes Infect 5: 419–427. 12737998 10.1016/s1286-4579(03)00050-9

[2] Dutra WO, Menezes CA, Magalhaes LM, Gollob KJ (2014) Immunoregulatory networks in human Chagas disease. Parasite Immunol. 10.1111/pim.12107 PMC4143493 24611805

[3] Costa GC, da Costa Rocha MO, Moreira PR, Menezes CA, Silva MR, et al (2009) Functional IL-10 gene polymorphism is associated with Chagas disease cardiomyopathy. J Infect Dis 199: 451–454. 10.1086/596061 19099482

[4] Villani FN, Rocha MO, Nunes Mdo C, Antonelli LR, Magalhaes LM, et al (2010) Trypanosoma cruzi-induced activation of functionally distinct alphabeta and gammadelta CD4- CD8- T cells in individuals with polar forms of Chagas' disease. Infect Immun 78: 4421–4430. 10.1128/IAI.00179-10 20696836 PMC2950361

[5] Souza PE, Rocha MO, Rocha-Vieira E, Menezes CA, Chaves AC, et al (2004) Monocytes from patients with indeterminate and cardiac forms of Chagas' disease display distinct phenotypic and functional characteristics associated with morbidity. Infect Immun 72: 5283–5291. 15322024 10.1128/IAI.72.9.5283-5291.2004 PMC517423

[6] Guedes PM, Gutierrez FR, Silva GK, Dellalibera-Joviliano R, Rodrigues GJ, et al (2012) Deficient regulatory T cell activity and low frequency of IL-17-producing T cells correlate with the extent of cardiomyopathy in human Chagas' disease. PLoS Negl Trop Dis 6: e1630 10.1371/journal.pntd.0001630 22545173 PMC3335880

[7] Magalhaes LM, Villani FN, Nunes Mdo C, Gollob KJ, Rocha MO, et al (2013) High interleukin 17 expression is correlated with better cardiac function in human Chagas disease. J Infect Dis 207: 661–665. 10.1093/infdis/jis724 23204182 PMC3611763

[8] Talvani A, Rocha MO, Barcelos LS, Gomes YM, Ribeiro AL, et al (2004) Elevated concentrations of CCL2 and tumor necrosis factor-alpha in chagasic cardiomyopathy. Clin Infect Dis 38: 943–950. 15034825 10.1086/381892

[9] Vago AR, Andrade LO, Leite AA, d'Avila Reis D, Macedo AM, et al (2000) Genetic characterization of Trypanosoma cruzi directly from tissues of patients with chronic Chagas disease: differential distribution of genetic types into diverse organs. Am J Pathol 156: 1805–1809. 10793092 10.1016/s0002-9440(10)65052-3 PMC1876933

[10] Zingales B, Andrade SG, Briones MR, Campbell DA, Chiari E, et al (2009) A new consensus for Trypanosoma cruzi intraspecific nomenclature: second revision meeting recommends TcI to TcVI. Mem Inst Oswaldo Cruz 104: 1051–1054. 20027478 10.1590/s0074-02762009000700021

[11] Zingales B, Miles MA, Campbell DA, Tibayrenc M, Macedo AM, et al (2012) The revised Trypanosoma cruzi subspecific nomenclature: rationale, epidemiological relevance and research applications. Infect Genet Evol 12: 240–253. 10.1016/j.meegid.2011.12.009 22226704

[12] Kawashita SY, Sanson GF, Fernandes O, Zingales B, Briones MR (2001) Maximum-likelihood divergence date estimates based on rRNA gene sequences suggest two scenarios of Trypanosoma cruzi intraspecific evolution. Mol Biol Evol 18: 2250–2259. 11719574 10.1093/oxfordjournals.molbev.a003771

[13] Breniere SF, Morochi W, Bosseno MF, Ordonez J, Gutierrez T, et al (1998) Trypanosoma cruzi genotypes associated with domestic Triatoma sordida in Bolivia. Acta Trop 71: 269–283. 9879736 10.1016/s0001-706x(98)00061-8

[14] Di Noia JM, Buscaglia CA, De Marchi CR, Almeida IC, Frasch AC (2002) A Trypanosoma cruzi small surface molecule provides the first immunological evidence that Chagas' disease is due to a single parasite lineage. J Exp Med 195: 401–413. 11854354 10.1084/jem.20011433 PMC2193624

[15] Freitas JM, Lages-Silva E, Crema E, Pena SD, Macedo AM (2005) Real time PCR strategy for the identification of major lineages of Trypanosoma cruzi directly in chronically infected human tissues. Int J Parasitol 35: 411–417. 15777917 10.1016/j.ijpara.2004.10.023

[16] Steindel M, Kramer Pacheco L, Scholl D, Soares M, de Moraes MH, et al (2008) Characterization of Trypanosoma cruzi isolated from humans, vectors, and animal reservoirs following an outbreak of acute human Chagas disease in Santa Catarina State, Brazil. Diagn Microbiol Infect Dis 60: 25–32. 17889480 10.1016/j.diagmicrobio.2007.07.016

[17] Pena DA, Eger I, Nogueira L, Heck N, Menin A, et al (2011) Selection of TcII Trypanosoma cruzi population following macrophage infection. J Infect Dis 204: 478–486. 10.1093/infdis/jir292 21742848

[18] Federici EE, Abelmann WH, Neva FA (1964) Chronic and Progressive Myocarditis and Myositis in C3h Mice Infected with Trypanosoma Cruzi. Am J Trop Med Hyg 13: 272–280. 14125879 10.4269/ajtmh.1964.13.272

[19] Andrade LO, Machado CR, Chiari E, Pena SD, Macedo AM (1999) Differential tissue distribution of diverse clones of Trypanosoma cruzi in infected mice. Mol Biochem Parasitol 100: 163–172. 10391378 10.1016/s0166-6851(99)90035-x

[20] Duz AL, Vieira PM, Roatt BM, Aguiar-Soares RD, Cardoso JM, et al (2014) The TcI and TcII Trypanosoma cruzi experimental infections induce distinct immune responses and cardiac fibrosis in dogs. Mem Inst Oswaldo Cruz 109: 1005–1013. 10.1590/0074-02760140208 25591108 PMC4325618

[21] Salgado FJ, Lojo J, Fernandez-Alonso CM, Vinuela J, Cordero OJ, et al (2002) Interleukin-dependent modulation of HLA-DR expression on CD4and CD8 activated T cells. Immunol Cell Biol 80: 138–147. 11940114 10.1046/j.1440-1711.2002.01055.x

[22] Aoki MP, Carrera-Silva EA, Cuervo H, Fresno M, Girones N, et al (2012) Nonimmune Cells Contribute to Crosstalk between Immune Cells and Inflammatory Mediators in the Innate Response to Trypanosoma cruzi Infection. J Parasitol Res 2012: 737324 10.1155/2012/737324 21869919 PMC3159004

[23] Abrahamsohn IA, Coffman RL (1996) Trypanosoma cruzi: IL-10, TNF, IFN-gamma, and IL-12 regulate innate and acquired immunity to infection. Exp Parasitol 84: 231–244. 8932773 10.1006/expr.1996.0109

[24] Cardillo F, Postol E, Nihei J, Aroeira LS, Nomizo A, et al (2007) B cells modulate T cells so as to favour T helper type 1 and CD8+ T-cell responses in the acute phase of Trypanosoma cruzi infection. Immunology 122: 584–595. 17635611 10.1111/j.1365-2567.2007.02677.x PMC2266037

[25] Holscher C, Kohler G, Muller U, Mossmann H, Schaub GA, et al (1998) Defective nitric oxide effector functions lead to extreme susceptibility of Trypanosoma cruzi-infected mice deficient in gamma interferon receptor or inducible nitric oxide synthase. Infect Immun 66: 1208–1215. 9488415 10.1128/iai.66.3.1208-1215.1998 PMC108035

[26] Silva JS, Morrissey PJ, Grabstein KH, Mohler KM, Anderson D, et al (1992) Interleukin 10 and interferon gamma regulation of experimental Trypanosoma cruzi infection. J Exp Med 175: 169–174. 1730915 10.1084/jem.175.1.169 PMC2119081

[27] Campos MA, Almeida IC, Takeuchi O, Akira S, Valente EP, et al (2001) Activation of Toll-like receptor-2 by glycosylphosphatidylinositol anchors from a protozoan parasite. J Immunol 167: 416–423. 11418678 10.4049/jimmunol.167.1.416

[28] Bafica A, Santiago HC, Goldszmid R, Ropert C, Gazzinelli RT, et al (2006) Cutting edge: TLR9 and TLR2 signaling together account for MyD88-dependent control of parasitemia in Trypanosoma cruzi infection. J Immunol 177: 3515–3519. 16951309 10.4049/jimmunol.177.6.3515

[29] Wang S, Chen L (2004) T lymphocyte co-signaling pathways of the B7-CD28 family. Cell Mol Immunol 1: 37–42. 16212919

[30] Souza PE, Rocha MO, Menezes CA, Coelho JS, Chaves AC, et al (2007) Trypanosoma cruzi infection induces differential modulation of costimulatory molecules and cytokines by monocytes and T cells from patients with indeterminate and cardiac Chagas' disease. Infect Immun 75: 1886–1894. 17283096 10.1128/IAI.01931-06 PMC1865727

[31] Roffe E, Rothfuchs AG, Santiago HC, Marino AP, Ribeiro-Gomes FL, et al (2012) IL-10 limits parasite burden and protects against fatal myocarditis in a mouse model of Trypanosoma cruzi infection. J Immunol 188: 649–660. 10.4049/jimmunol.1003845 22156594 PMC3253255

[32] Silva JS, Twardzik DR, Reed SG (1991) Regulation of Trypanosoma cruzi infections in vitro and in vivo by transforming growth factor beta (TGF-beta). J Exp Med 174: 539–545. 1908509 10.1084/jem.174.3.539 PMC2118925

[33] Gomes JA, Molica AM, Keesen TS, Morato MJ, de Araujo FF, et al (2014) Inflammatory mediators from monocytes down-regulate cellular proliferation and enhance cytokines production in patients with polar clinical forms of Chagas disease. Hum Immunol 75: 20–28. 10.1016/j.humimm.2013.09.009 24071371

[34] Gravina HD, Antonelli L, Gazzinelli RT, Ropert C (2013) Differential use of TLR2 and TLR9 in the regulation of immune responses during the infection with Trypanosoma cruzi. PLoS One 8: e63100 10.1371/journal.pone.0063100 23650544 PMC3641106

[35] Carvalho LP, Pearce EJ, Scott P (2008) Functional dichotomy of dendritic cells following interaction with Leishmania braziliensis: infected cells produce high levels of TNF-alpha, whereas bystander dendritic cells are activated to promote T cell responses. J Immunol 181: 6473–6480. 18941238 10.4049/jimmunol.181.9.6473 PMC2754122

[36] Reis DD, Jones EM, Tostes S Jr., Lopes ER, Gazzinelli G, et al (1993) Characterization of inflammatory infiltrates in chronic chagasic myocardial lesions: presence of tumor necrosis factor-alpha+ cells and dominance of granzyme A+, CD8+ lymphocytes. Am J Trop Med Hyg 48: 637–644. 8517482 10.4269/ajtmh.1993.48.637

[37] Fuenmayor C, Higuchi ML, Carrasco H, Parada H, Gutierrez P, et al (2005) Acute Chagas' disease: immunohistochemical characteristics of T cell infiltrate and its relationship with T. cruzi parasitic antigens. Acta Cardiol 60: 33–37. 15779849 10.2143/AC.60.1.2005046

[38] Dutra WO, Martins-Filho OA, Cancado JR, Pinto-Dias JC, Brener Z, et al (1994) Activated T and B lymphocytes in peripheral blood of patients with Chagas' disease. Int Immunol 6: 499–506. 8018591 10.1093/intimm/6.4.499

[39] Kumar S, Tarleton RL (1998) The relative contribution of antibody production and CD8+ T cell function to immune control of Trypanosoma cruzi. Parasite Immunol 20: 207–216. 9651921 10.1046/j.1365-3024.1998.00154.x

[40] Todeschini AR, Nunes MP, Pires RS, Lopes MF, Previato JO, et al (2002) Costimulation of host T lymphocytes by a trypanosomal trans-sialidase: involvement of CD43 signaling. J Immunol 168: 5192–5198. 11994475 10.4049/jimmunol.168.10.5192

[41] da Matta Guedes PM, Gutierrez FR, Maia FL, Milanezi CM, Silva GK, et al (2010) IL-17 produced during Trypanosoma cruzi infection plays a central role in regulating parasite-induced myocarditis. PLoS Negl Trop Dis 4: e604 10.1371/journal.pntd.0000604 20169058 PMC2821906

[42] Miyazaki Y, Hamano S, Wang S, Shimanoe Y, Iwakura Y, et al (2010) IL-17 is necessary for host protection against acute-phase Trypanosoma cruzi infection. J Immunol 185: 1150–1157. 10.4049/jimmunol.0900047 20562260

[43] Erdmann H, Rossnagel C, Bohme J, Iwakura Y, Jacobs T, et al (2013) IL-17A promotes macrophage effector mechanisms against Trypanosoma cruzi by trapping parasites in the endolysosomal compartment. Immunobiology 218: 910–923. 10.1016/j.imbio.2012.10.005 23182712

[44] Padilla AM, Bustamante JM, Tarleton RL (2009) CD8+ T cells in Trypanosoma cruzi infection. Curr Opin Immunol 21: 385–390. 10.1016/j.coi.2009.07.006 19646853 PMC2735075

[45] Higuchi Mde L, Gutierrez PS, Aiello VD, Palomino S, Bocchi E, et al (1993) Immunohistochemical characterization of infiltrating cells in human chronic chagasic myocarditis: comparison with myocardial rejection process. Virchows Arch A Pathol Anat Histopathol 423: 157–160. 7901937 10.1007/BF01614765

[46] Muller U, Sobek V, Balkow S, Holscher C, Mullbacher A, et al (2003) Concerted action of perforin and granzymes is critical for the elimination of Trypanosoma cruzi from mouse tissues, but prevention of early host death is in addition dependent on the FasL/Fas pathway. Eur J Immunol 33: 70- 12594834 10.1002/immu.200390009

[47] Adesse D, Iacobas DA, Iacobas S, Garzoni LR, Meirelles Mde N, et al (2010) Transcriptomic signatures of alterations in a myoblast cell line infected with four distinct strains of Trypanosoma cruzi. Am J Trop Med Hyg 82: 846–854. 10.4269/ajtmh.2010.09-0399 20439965 PMC2861399

