# Supplementary figures and images for: Differential Activation of Human Monocytes and Lymphocytes by Distinct Strains of Trypanosoma cruzi
Source: PLoS Negl Trop Dis. 2015 Jul 6;9(7):e0003816. doi: 10.1371/journal.pntd.0003816 (PMC4492932; doi:10.1371/journal.pntd.0003816)

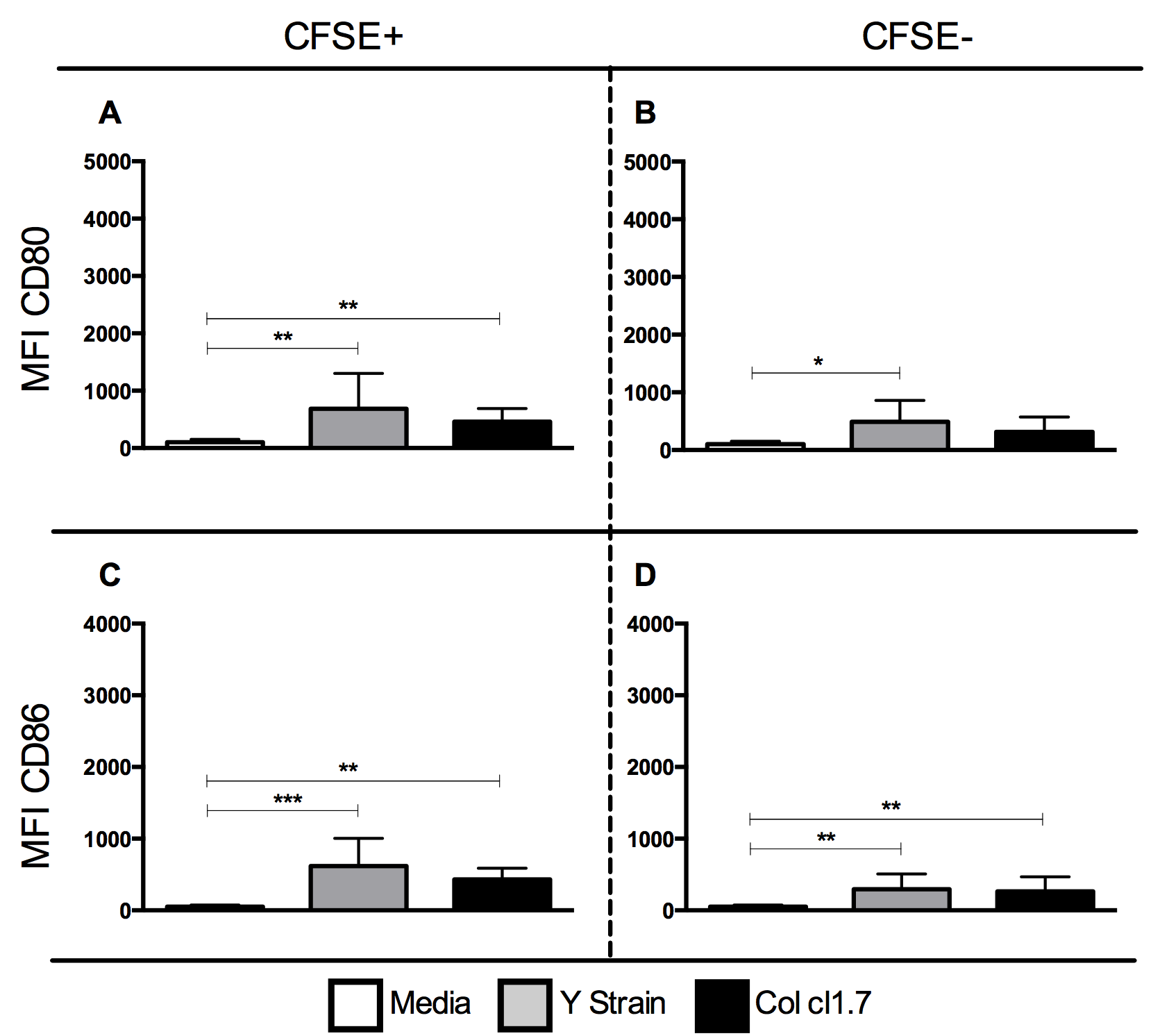

Supplement: S1 Fig — Results are expressed as average ± standard deviation. Determination of mean intensity of (A) CD80 in CFSE+ monocytes after 15 hours of culture; (B) CD80 in CFSE- monocytes after 15 hours of culture; (C) CD86 in CFSE+ monocytes after 15 hours of culture; (D) CD86 in CFSE- monocytes after 15 hours of culture. (TIFF) [file pntd.0003816.s001.tiff]

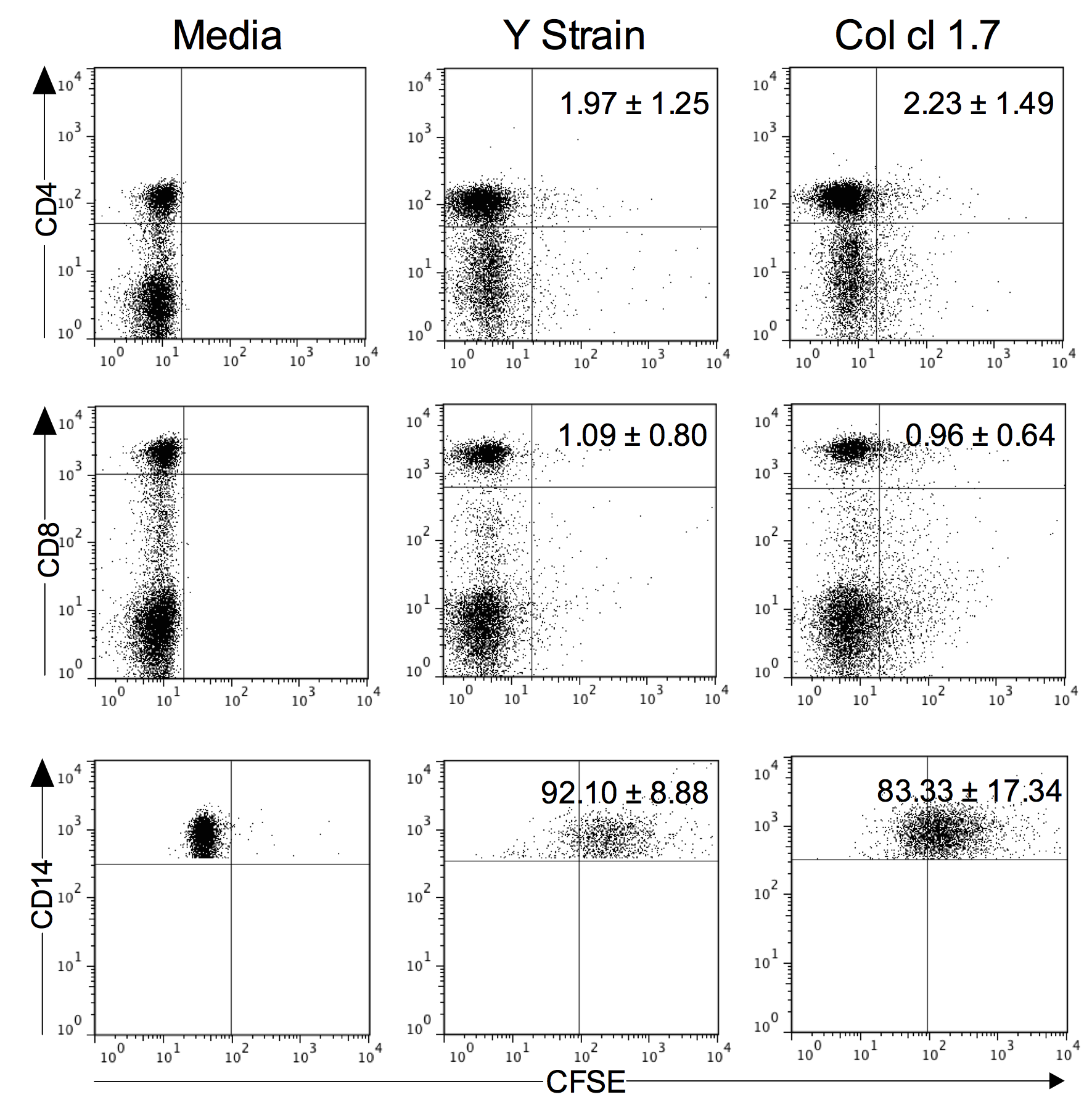

Supplement: S2 Fig — Representative dot-plots of the analysis of infection of CD4 and CD8+ cells (gated on lymphocytes) and CD14+ cells (gated on monocytes) by the different isolates. The first three panels show media control and the others show Y strain and Col cl1.7, for each cell population. The figure shows that CD4+ and CD8+ cells display very low infection by both strains and that monocytes are the main infected cell population. Moreover, no differences were observed when comparing monocyte infectivity by Y and Col cl1.7 isolates (quantitative data shown in Fig 1C and 1D). (TIFF) [file pntd.0003816.s002.tiff]
